# Patterns of Care, Prognostic Factors, and Survival Outcomes for Patients of Cervical Carcinoma: A Study From North India

**DOI:** 10.7759/cureus.71824

**Published:** 2024-10-19

**Authors:** Purnima Thakur, Kaalindi Singh, Vineet Kumar, Manish Gupta, Shabnam Thakur

**Affiliations:** 1 Department of Radiation Oncology, Indira Gandhi Medical College, Shimla, IND; 2 Department of Radiotherapy, Shri Lal Bahadur Shastri Government Medical College and Hospital, Mandi, IND; 3 Department of Community Medicine, Indira Gandhi Medical College, Shimla, IND

**Keywords:** carcinoma cervix, north india, pattern of care, prognostic factors, survival outcomes

## Abstract

Objectives: Cervical cancer is the second most common malignancy among Indian women after breast cancer. This study was undertaken to determine the pattern of care, long-term survival outcomes, and prognostic factors for cervical cancer patients treated at a tertiary care cancer centre in North India.

Methods: Ten-year data was retrieved for 435 stage I-IVA carcinoma cervix patients treated between 2009 and 2019. A sociodemographic profile of the patients, treatment methods, and conditions at follow-up were obtained.

Statistical analysis: Data was analyzed using Stata 15.1 (StataCorp LLC, College Station, Texas, United States). Qualitative variables were shown as frequencies/percentages, and quantitative as means/standard deviations. Key endpoints were overall, disease-free, and locoregional survival. Kaplan-Meier curves represented survival. Cox regression models assessed parameter influence. Statistical significance was set at p < 0.05, with hazard ratios and 95% confidence intervals reported.

Results: Fourteen (3.2%) patients underwent surgery; of the patients who underwent surgery, except two patients who underwent surgery alone, all patients received external beam radiotherapy (EBRT) followed by EBRT (26.4%) or brachytherapy boost (72.4%). Seventy-one per cent of patients received more than four cycles of concurrent chemotherapy. The median overall survival (OS) was 42.04 (0.25-171) months, and the median disease-free survival (DFS) was 32.85 months (1-171) months. The median overall survival was 58.76%, and the two-year and five-year overall survival percentages were 81.34% and 64.52%, respectively. The median time to locoregional relapse and distant metastasis was 35 (1-171) and 37.6 (1-171) months, respectively. Bilateral parametrial involvement was a predictor of poor OS (p = 0.039), DFS (p = 0.039), and locoregional failure-free survival (LFFS) (p = 0.020). Age less than 50 years was a predictor of worse DFS (p = 0.034) and LFFS (p = 0.005), while paraaortic nodal involvement was a predictor of worse DFS (p = 0.045). Grade III tumours neared numerical significance (p = 0.051) for worse distant metastases-free survival.

Conclusion: Bilateral parametrial involvement was the most significant factor for OS, DFS, and LFFS. Paraaortic nodal involvement predicted poor DFS and young age was associated with poor DFS and LFFS.

## Introduction

Cervical cancer is the second most common gynaecological malignancy among Indian women, as per Globocan 2020 [1]. Worldwide, it has a five-year prevalence of 19,48,521 cases and an incidence of 6,62,301 new cases per year. As per Indian Council of Medical Research (ICMR) data, carcinoma cervix has an incidence of 79,103 cases per year in India and a cumulative risk of 11.1 between ages 0-74 years, and the burden is projected to increase to 85,241 cases by 2025 [2]. Lower socioeconomic status, poor personal hygiene, multiple and early childbirths, human papillomavirus infection, promiscuous sexual behaviour, and sexually transmitted diseases are some of the risk factors predisposing to carcinoma cervix [3-6]. The treatment is primarily dictated by the International Federation of Gynaecology and Obstetrics (FIGO) stage of the disease and the patient's general condition. Concurrent chemoradiation followed by brachytherapy remains the standard of care in managing locally advanced carcinoma cervix [7-11]. Surgery alone is often considered in non-bulky stage I disease [12,13]. Adding image guidance to radiotherapy has improved outcomes by improving dose delivery to the target tissue and reducing toxicity to normal surrounding structures [14,15].

FIGO staging, with continuous updation, has stood the test of time [16]. Besides the stage, several other treatment and socio-demographic factors impact the disease outcomes, which may be unique to regions and communities [17-19]. Determining these helps modify policies at regional and institutional levels to enhance outcomes. In low-middle income countries like India, where women often feel shame and embarrassment in reporting to hospitals, delays in diagnosis and treatment initiation increase morbidity and mortality.

In this study, we undertook to determine the patterns of care, survival outcomes, and factors affecting them for carcinoma cervix patients treated at a tertiary cancer care centre in North India. The sociodemographic and disease-related factors mirror rural India, primarily addressing the needs of its rural population, given that around 90% of the residents in Himachal Pradesh, India, who report to our centre come from rural regions. Moreover, these findings hold significance for the developing parts of the world [20].

## Materials and methods

This retrospective study was conducted at a tertiary care cancer centre, Indira Gandhi Medical College, in North India. The treatment records of all carcinoma cervix patients radically treated between 2009 and 2019 were retrieved. The socio-demographic characteristics, staging, treatment details, and follow-ups were documented on a pre-designed proforma.

Inclusion criteria

Stage I-IVA carcinoma cervix patients, as per the FIGO staging system, 2009 [21], with biopsy-proven malignancy with no prior history of pelvic radiation were included.

Exclusion criteria

Upfront metastatic carcinoma cervix patients and those with a history of second malignancy were excluded. All the collected data was compiled into a tabular form for evaluation.

Statistical analysis

Data was analyzed using Stata software version 15.1 (StataCorp LLC, College Station, Texas, United States). Qualitative variables were presented as frequencies and their percentages, whereas quantitative data was described as means and standard deviations. Endpoints in this study were overall survival, disease-free survival (DFS), locoregional progression, and distant metastasis-free survival. All the survivals were calculated from the date of the start of treatment. Survivals were represented by the Kaplan-Meier curves. Univariate and multivariate Cox proportional hazards regression models were used to evaluate the influence of parameters on different survivals. Each parameter was assessed by univariate analysis. All variables were included for multivariate analysis to estimate the hazard ratio and 95% confidence interval. Statistical significance was defined as p-values <0.05.

## Results

Patient characteristics

The median age of patients presenting to our clinic was 52 (range: 28-83) years. The average haemoglobin level was 10.97+/- 1.95 mg/dl. Only 14 (3.2%) patients had surgery as part of their initial management; the remaining underwent radical radiotherapy with or without concurrent chemotherapy. Detailed patient and disease characteristics are summarised in Table 1.

**Table 1:** Patient and disease characteristics

Patient characteristic		Number (N)	Percentage (%)
Haemoglobin	<10 mg/dl	139	31.95
	>10mg/dl	296	68.04
	Total	435	
Histology	Squamous cell carcinoma	406	93.3
	Adenocarcinoma	21	4.8
	Adenosquamous carcinoma	6	1.38
	Clear cell carcinoma	1	0.2
	Glossy cell carcinoma	1	0.2
	Total	435	
Grade	Well-differentiated	80	18.4
	Moderately differentiated	251	57.7
	Poorly differentiated	44	10.1
	Not known	60	13.8
	Total	435	
Stage	I	7	1.6
	IIA	12	2.8
	IIB	278	63.9
	IIIA	9	2.1
	IIIB	126	28.96
	IVA	3	0.7
	Total	435	
Parametrial involvement	Unilateral	125	28.7
	Bilateral	286	65.7
	Not known	24	5.5
	Total	435	
Parametrial Bulk	Minimal	49	11.3
	Less than half	72	16.6
	More than half	291	66.89
	Not known	23	5.3
	Total	435	
Tumour mass	Less than 4 cm	100	22.98
	More than 4 cm	320	73.6
	Not known	15	3.4
	Total	435	
Pelvic side wall	Unilateral	100	22.98
	Bilateral	36	8.3
	Not known	299	68.7
	Total	435	
Vaginal involvement	Yes	214	49.2
	No	221	51.1
	Total	435	
Lymph nodes	No involved lymph nodes	275	63.2
	Pelvic lymph nodes	112	25.7
	Paraaortic lymph nodes	48	11
	Total	435	

Pre-treatment workup

Complete history and physical examination were done for all patients with clinical staging. All patients had histopathological confirmation with cervical biopsy. Complete hemograms and blood biochemistry were done for all patients. Urine routine microscopy was done in the majority of patients. For staging purposes, Computed tomography (CT) of the abdomen and pelvis and chest X-ray were done for all patients to determine the locoregional extent of the disease and exclude distant metastases. Magnetic resonance imaging (MRI) was done in selected patients if clinically indicated.

Treatment characteristics

The mean time to treatment initiation (TTI) from the date of diagnosis was 2.9 weeks (range: 0 - 44.4 weeks). The median overall treatment time (external beam radiotherapy (EBRT) + brachytherapy) was 8.7 weeks (range: 6 - 72 weeks) (Table 2).

**Table 2:** Treatment characteristics #: fraction; SFRT: single-fraction radiotherapy; @: at the rate; LDR: low dose rate; HDR: high dose rate; ICBT: intracavitary brachytherapy; MUPIT: Martinez Universal Perineal Interstitial Template; BED: biologically equivalent dose; EBRT: external beam radiotherapy

Treatment characteristic		Number (N)	Percentage (%)
Time to treatment initiation	< 4 weeks	348	80
	> 4 weeks	87	20
	Total	435	
Overall treatment time	< 8 weeks	155	35.6
	> 8 weeks	276	63.4
	Not available	4	0.91
	Total	435	
Treatment interruption	Yes	6	1.4
	No	427	98.1
	Not available	2	0.46
	Total	435	
Duration of treatment interruption	< 1 week	3	0.69
	> 1 week	3	0.69
	Total	6	
Radiotherapy dose	50Gy/25#/5week	258	59.3
	46Gy/23#/ 4.5week	124	28.5
	35Gy/15#/3weeks+SFRT 10Gy	29	6.67
	50Gy/25#/4 weeks	16	3.7
	20Gy/10#/2 weeks followed by 30Gy/20#/2 weeks @ 1.5 Gy twice daily	6	1.37
	Not available	2	0.46
	Total	435	
Pelvic boost	Brachytherapy	315	72.4
	Supplement EBRT	115	26.4
	No Boost	5	1.14
	Total	435	
Type of brachytherapy	LDR	77	17.7
	HDR	241	55.4
	ICBT	316	72.6
	MUPIT	2	0.45
Dose of brachytherapy boost	7Gy x 3#	138	31.7
	9Gy x 2#	101	23.2
	9.5Gy x 2#	2	0.46
	8Gy x 3#	1	0.23
	4.5 x 4#	1	0.23
	35Gy with LDR	76	17.5
Total BED delivered	>85 Gy	322	74
	<85 Gy	112	25.7
Concurrent chemotherapy	No chemotherapy	62	14.2
	> 4 cycles	361	82.9
	< 4 cycles	12	2.8
	Total	435	

Surgery

Fourteen patients (3.2 %) underwent surgery, 13 upfront and one patient with stage II disease for relapse. On pathological evaluation, the stagewise distribution was as follows: stage I-1 (7.7%), stage II-9 (69.2%), stage III-4 (30.8%), and stage IV-0. Twelve out of 14 patients received adjuvant concurrent chemoradiation followed by a brachytherapy boost. 

Radiotherapy 

The planning was done on a conventional X-ray-based simulator. Patients were treated in the supine position with heel lock and knee rest. A vaginal marker was placed for the lower extent of the disease. Two-field and four-field techniques were used depending on patient separation. In the anterior and posteroanterior (AP and PA) fields, the superior extent of the portal was placed at the L4-L5 vertebral junction; in patients with involved paraaortic lymph nodes, the superior border was placed at the T11-12 junction. The lateral border was placed 2 centimetres lateral to the broadest portion of the pelvic cavity. Inferiorly, the border was placed at 2 cm distal to the lower extent of disease or the inferior border of obturator foramen, whichever was lower. For lateral fields, the posterior border was placed at the junction of S2-S3 in patients with stage II disease, and in stage III patients, the entire sacral hollow was included; the anterior border was placed at the anterior surface of the pubic symphysis. The superior and inferior borders remained the same as AP/PA fields. Cobalt-60 was used for the delivery of radiation therapy.

Boost was delivered to gross disease with brachytherapy (72.4%) or supplement EBRT (26.4%). Although brachytherapy remains the standard of care as a modality of boost, EBRT had to be used for boost in the interim period between decommissioning of old brachytherapy equipment and installation of new brachytherapy equipment, which was prolonged due to logistic issues. Only those patients who could not afford to go to other centres for brachytherapy treatment were given an EBRT boost. The radiation field of EBRT boost was limited to the initial gross disease, and a dose of 20 Gy in 10 fractions was delivered to reduce bladder and rectal toxicity. In patients receiving brachytherapy boost, X-ray-based brachytherapy planning was done, and bilateral point A doses were documented. Also, bladder point and rectal point doses were documented as per the International Commission on Radiation Units and Measurements (ICRU) report 38 for assessment of dose to organs at risk. Most patients (98.1%) completed EBRT treatment without any interruption.

In patients receiving a high dose-rate (HDR) brachytherapy boost, it was delivered by HDR microSelectron (Elekta AB, Stockholm, Sweden), using a 192 Ir source and Fletcher Williamson Asia Pacific applicators (Elekta AB, Stockholm, Sweden). The mean biologically effective dose (BED) to tumour tissue was 95.7 Gy (range 55.2 to 112.1 Gy) and the equivalent dose in two Gy fractions (EQD 2) to point A was 79.75 Gy. 

Concurrent weekly chemotherapy was delivered weekly at a dose of 40 mg/m2. At least one cycle of concurrent chemotherapy was delivered in 373 (85.7%) patients, out of which 361 (83%) received more than four cycles. In the remaining patients, chemotherapy was omitted due to poor tolerance to chemoradiotherapy (CRT). All patients planned for radiotherapy completed treatment.

Survival outcome

The median overall survival was 42.2 (range: 1 - 171) months. The median overall survival, two-year, and five-year survival were 58.76%, 81.34%, and 64.52%, respectively. Median DFS was 33.1 (range 1 - 171) months. The median time to locoregional failure and distant metastases was 35 (range: 1 - 171) months and 37.6 (range: 1 - 171) months, respectively.

A total of 150 (34.5%) patients experienced relapse; of these, 95 (63.3%) were only local, 10 (6.7%) had both local and distant relapse, and 44 (29.3%) had only distant metastasis.

The most common site of distant metastasis was bone, with lumbar vertebrae and pelvic bones (18 patients, 33.3%) being the most affected. The second most common site of metastases was the lung in 16 (29.6%), followed by the liver in 15 (27.8%) patients. Paraaortic nodal relapse occurred in eight (14.8%) patients; isolated paraaortic nodal relapse occurred in five (9.2%) patients. Ascites occurred in five (9.2%) patients. However, the nature of ascites, that is, malignant versus non-malignant, was not confirmed. Brain metastasis occurred in two (3.7%) patients out of 54 who experienced distant metastasis.

Factors affecting disease outcome

The effects of several disease- and treatment-related factors on disease outcomes were analysed. Univariate and multivariate analysis results on overall survival, DFS, locoregional relapse-free survival, and distant metastasis-free survival are summarised in tables.

On univariate analysis, overall treatment time of more than eight weeks (p-value = 0.02), stage >/=IIIA (p-value = 0.001), bilateral parametrial involvement (p-value = 0.006), and presence of pelvic lymph nodes were all found to be significant for lower overall survival. On multivariate analysis, only bilateral parametrial involvement was predictive of poor overall survival (p-value = 0.039) (Table 3).

**Table 3:** Factors affecting overall survival, univariate and multivariate analyses *p-value<0.05 is considered significant

Characteristic	Univariate analysis		Multivariate analysis	
	HR	p-value	HR (95% CI)	p-value
Age (< 50 years vs ≥ 50 years)	1.02	0.879	1.07 (0.78- 1.47)	0.674
Pre-treatment haemoglobin (< 10 gm/dl vs ≥ 10 gm/dl)	0.87	0.397	0.83 (0.59-1.16)	0.277
Time to treatment initiation ( > 4 weeks vs ≤ 4 weeks)	1.30	0.174	1.24 (0.84-1.32)	0.269
Lymph node pelvic (present vs none)	1.44	0.017*	1.08 (0.74-1.57)	0.684
Lymph node paraaortic (present vs none)	1.50	0.060	1.20 (0.73-1.97)	0.464
Overall treatment time (> 8 weeks vs ≤ 8 weeks)	1.46	0.020*	1.09 (0.79-1.58)	0.664
Histology (non-squamous vs squamous)	1.38	0.266	1.41 (0.75-2.65)	0.286
Grade (high vs low and intermediate)	1.31	0.262	1.37 (0.85-2.20)	0.199
Stage (≥ IIIA vs < IIIA)	1.65	0.001*	1.36 (0.99-1.89)	0.062
Para (bilateral vs unilateral)	1.60	0.006*	1.47 (1.02-2.13)	0.039*
Boost (supplement radiotherapy vs brachytherapy)	1.84	<0.001*	1.45 (0.69-3.05)	0.328
Biologically effective dose (BED) (< 85 Gy vs ≥ 85 Gy)	0.57	<0.001*	0.88 (0.42-1.85)	0.728

Poor DFS was associated with the presence of pelvic and paraaortic lymph nodes, bilateral parametrial involvement, use of EBRT for boost, stage III and above, BED </=85 Gy and overall treatment time of more than eight weeks on univariate analysis. On multivariate analysis, only paraaortic nodal involvement and bilateral parametrial involvement were found to be significant predictors of worse DFS (Table 4).

**Table 4:** Univariate and multivariate analyses for disease-free survival *p-value <0.05 considered significant

Characteristic	Univariate analysis		Multivariate analysis	
	HR	p-value	HR (95% CI)	p-value
Age (< 50 years vs ≥ 50 years)	1.24	0.194	1.45 (1.03-2.06)	0.034*
Pre-treatment haemoglobin (< 10 gm/dl vs ≥ 10 gm/dl)	0.86	0.404	0.73 (0.81-1.86)	0.328
Time to treatment initiation ( > 4 weeks vs ≤ 4 weeks)	1.23	0.294	1.23 (0.81-1.86)	0.328
Lymph node pelvic (present vs none)	1.64	0.003*	1.10 (0.73-1.65)	0.645
Lymph node paraaortic (present vs none)	1.92	0.003*	1.71 (1.01-2.89)	0.045*
Overall treatment time (> 8 weeks vs ≤ 8 weeks)	1.52	0.021*	1.18 (0.79-1.79)	0.420
Histology (non-squamous vs squamous)	1.45	0.197	1.69 (0.90-3.18)	0.103
Grade (high vs low and intermediate)	1.28	0.337	1.32 (0.78-2.25)	0.302
Stage (≥ IIIA vs < IIIA)	1.59	0.006*	1.26 (0.88-1.81)	0.205
Para (bilateral vs unilateral)	1.69	0.008*	1.54 (1.02-2.32)	0.039*
Boost (supplement radiotherapy vs brachytherapy)	1.95	<0.001*	1.66 (0.73-3.80)	0.228
Biologically effective dose (BED) (< 85 Gy vs ≥ 85 Gy)	0.55	0.001*	0.95 (0.41-2.20)	0.913

Locoregional failure-free survival was adversely affected by the presence of pelvic and paraaortic lymph nodes( p-value = 0.002 and 0.039, respectively), overall treatment time of more than eight weeks, stage >/= IIIA (p-value 0.001), bilateral parametrial involvement, use of EBRT for boost and BED < 85 Gy (p-value<0.001), on univariate analysis. On the correction for all factors on multivariate analysis, age < 50 years (p-value = 0.005) and bilateral parametrial involvement (p-value = 0.019) were associated with worse locoregional failure-free survival (Table 5).

**Table 5:** Univariate and multivariate analyses for locoregional failure-free survival *p-value <0.05 considered significant

Characteristic	Univariate analysis		Multivariate analysis	
	HR	p-value	HR (95% CI)	p-value
Age (< 50 years vs ≥ 50 years)	1.396819	0.067	1.70 (1.17-2.48)	0.005*
Pre-treatment haemoglobin (< 10 gm/dl vs ≥ 10 gm/dl)	0.8494942	0.413	0.74 (0.49-1.11)	0.144
Time to treatment initiation (> 4 weeks vs ≤ 4 weeks)	1.18295	0.456	1.22 (0.77-1.92)	0.400
Lymph node pelvic (present vs none)	1.741701	0.002*	1.21 (0.79-1.86)	0.388
Lymph node paraaortic (present vs none)	1.676086	0.039*	1.37 (0.77-2.43)	0.284
Overall treatment time (> 8 weeks vs ≤ 8 weeks)	1.620051	0.018*	1.25 (0.79-1.96)	0.338
Histology (non-squamous vs squamous)	1.030268	0.935	1.42 (0.68-2.97)	0.347
Grade (high vs low and intermediate)	0.9550389	0.884	1.04 (0.55-1.95)	0.906
Stage (≥ IIIA vs < IIIA)	1.819146	0.001*	1.39 (0.95-2.05)	0.093
Para (bilateral vs unilateral)	1.976934	0.003*	1.75 (1.10-2.80)	0.019*
Boost (supplement radiotherapy vs brachytherapy)	2.134365	<0.001*	2.04 (0.90-4.67)	0.090
Biologically effective dose (BED) (< 85 Gy vs ≥ 85 Gy)	0.5077717	<0.001*	1.14 (0.49-2.63)	0.757

When the impact of predefined variables on distant metastasis-free survival was analysed, none of the factors reached significance on univariate analysis. On multivariate analysis, high tumour grade was borderline significant with a p-value of 0.051 (Table 6).

**Table 6:** Univariate and multivariate Cox regression analyses for distant metastasis-free survival *p-value<0.05 is considered significant

Characteristic	Univariate analysis		Multivariate analysis	
	HR	p-value	HR (95% CI)	p-value
Age (< 50 years vs ≥ 50 years)	0.70	0.229	0.74 (0.39-1.40	0.352
Pre-treatment haemoglobin (< 10 gm/dl vs ≥ 10 gm/dl)	0.71	0.272	0.55 (0.28-1.12)	0.099
Time to treatment initiation (> 4 weeks vs ≤ 4 weeks)	1.69	0.094	1.64 (0.84- 3.21)	0.147
Lymph node pelvic (present vs none)	1.13	0.665	0.79 (0.37- 1.71)	0.553
Lymph node paraaortic (present vs none)	1.57	0.239	1.70 (0.63- 4.60)	0.297
Overall treatment time (> 8 weeks vs ≤ 8 weeks)	1.61	0.128	1.32 (0.64-2.70)	0.451
Histology (non-squamous vs squamous)	1.64	0.292	1.01 (0.30-3.39)	0.982
Grade (high vs low and intermediate)	2.11	0.043*	2.17 (0.99- 4.73)	0.051
Stage (≥ IIIA vs < IIIA)	1.25	0.440	1.15 (0.61-2.19)	0.664
Para (bilateral vs unilateral)	1.22	0.535	1.14 (0.59-2.22)	0.697
Boost (supplement radiotherapy vs brachytherapy)	1.27	0.434	0.68 (0.12-3.79)	0.663
Biologically equivalent dose (BED) (< 85 Gy vs ≥ 85 Gy)	0.84	0.581	0.60 (0.11-3.35)	0.563

**Figure 1:**
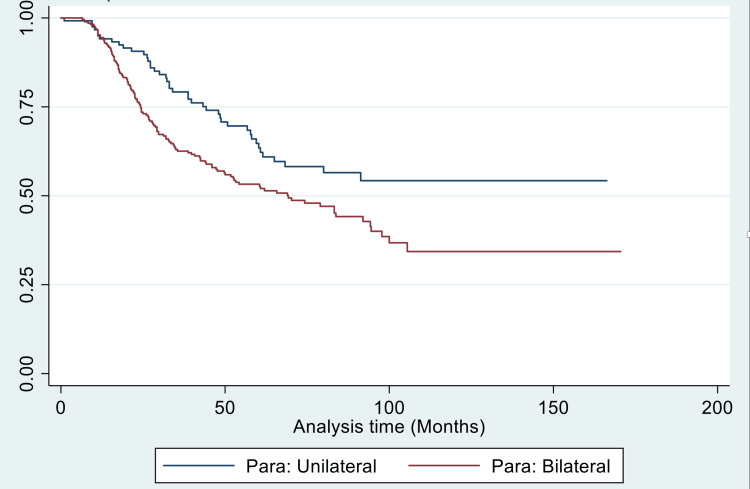
Kaplan-Meier curve showing overall survival (in months) in unilateral and bilateral parametrial involvement

On multivariate analysis, bilateral parametrial involvement alone significantly predicted worse overall survival (p-value = 0.039) (Figure 1).

**Figure 2:**
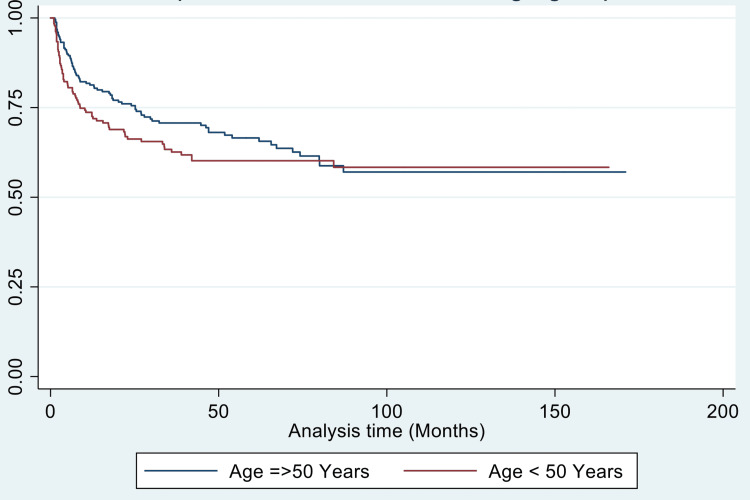
Kaplan-Meier curve for disease-free survival (in months) in two age groups: >/= 50 years versus < 50 years

**Figure 3:**
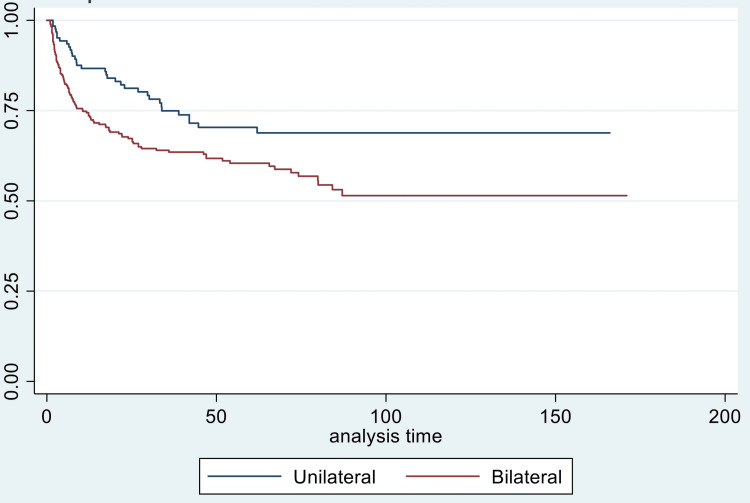
Kaplan-Meier curve for disease-free survival (in months) in two groups of parametrium involvement: unilateral versus bilateral

**Figure 4:**
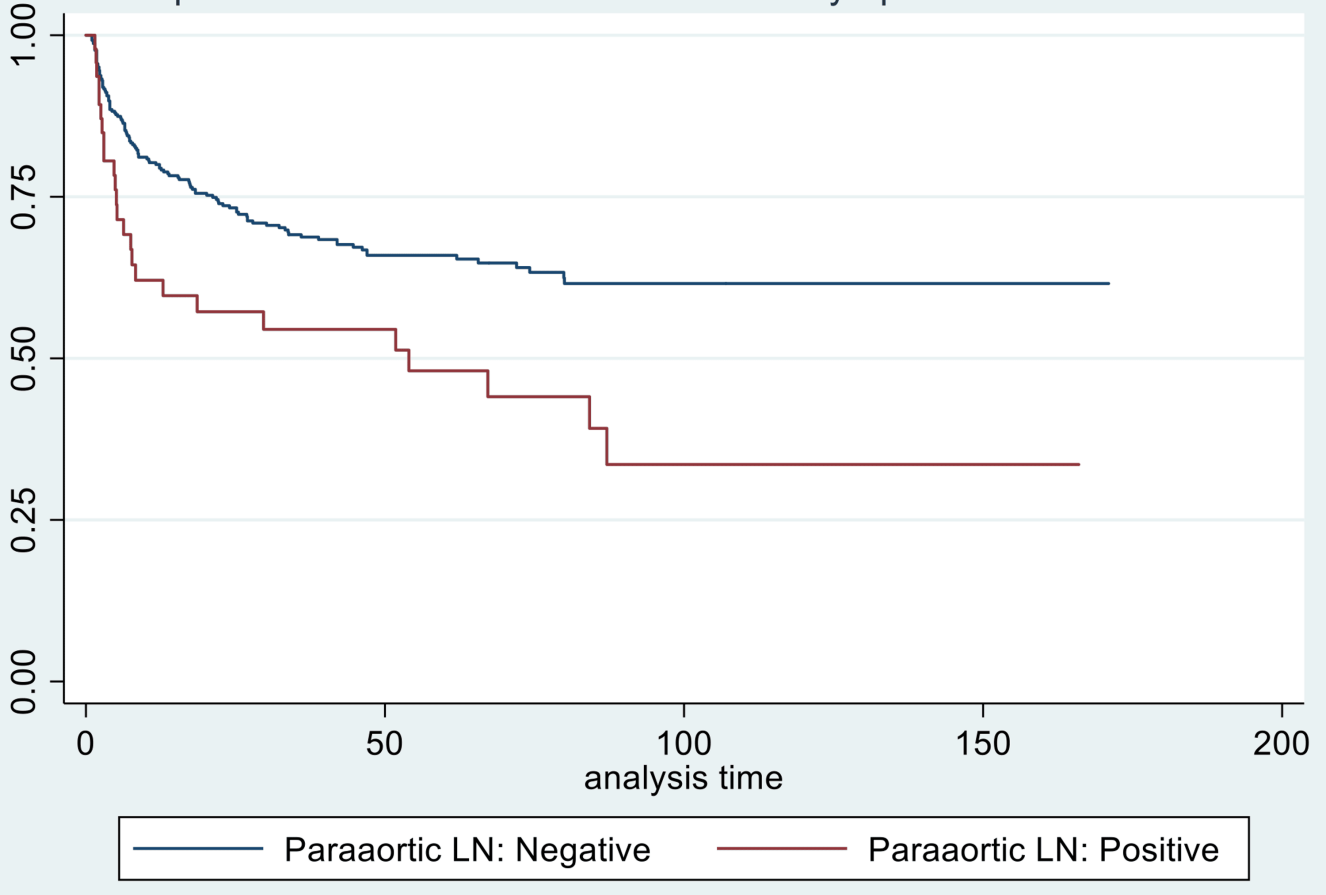
Kaplan-Meier curve comparing disease-free survival (in months) in patients with and without paraaortic lymph node (LN) involvement

For disease-free survival, young age (p-value = 0.034), bilateral parametrical involvement (p-value = 0.039), and paraaortic nodal involvement (p = 0.045) were significant predictors of worse DFS (Figures 2-4).

**Figure 5:**
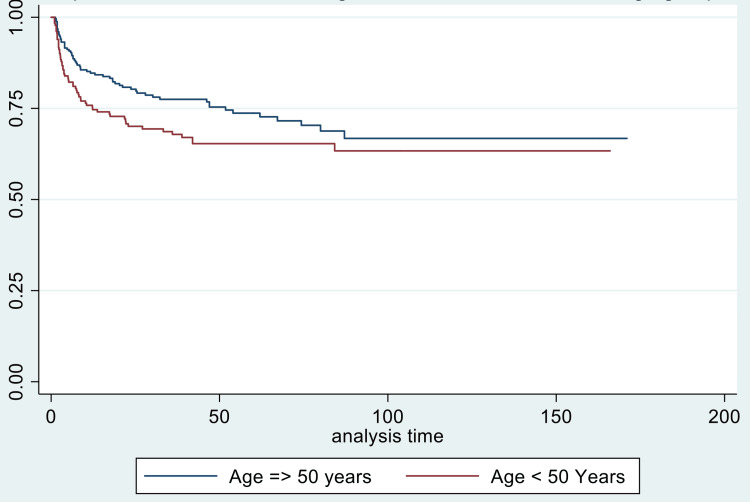
Kaplan-Meier curve showing locoregional failure-free survival (in months) in age groups >/= 50 years versus < 50 years

**Figure 6:**
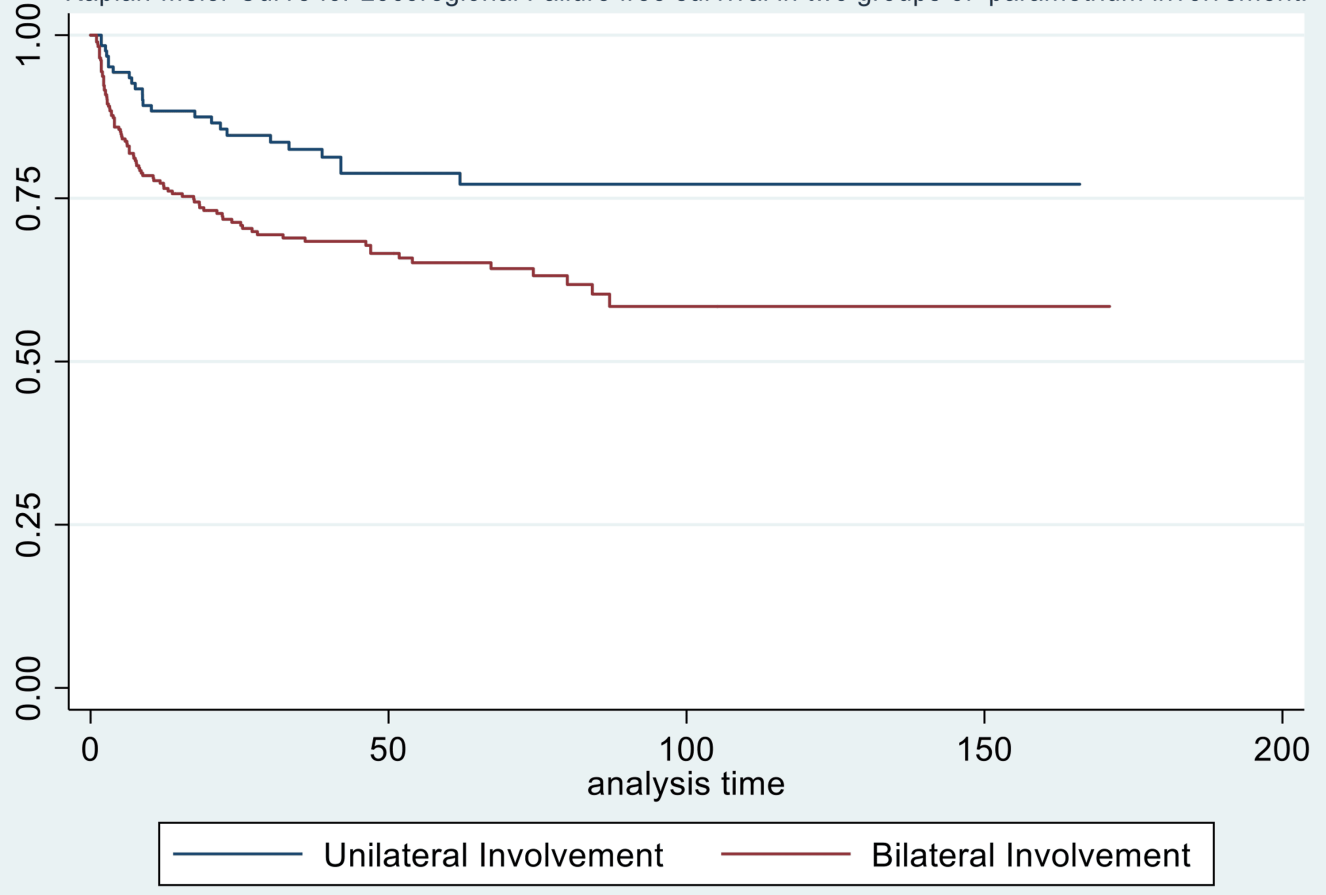
Kaplan-Meier curve showing locoregional failure-free survival (in months) in patients with unilateral versus bilateral parametrial involvement

Also, for locoregional recurrence, bilateral parametrial involvement (p-value = 0.020) and age < 50 years(p-value = 0.005) were found to be associated with worse outcomes (Figures 5, 6).

## Discussion

In this retrospective study, 10-year data from 435 stage I-IVA patients was analysed to understand the patterns of care, survival outcomes and the effect of different disease and treatment-related factors on outcomes. As per FIGO 2009 staging, only seven (1.6%) patients presented in stage I, and all remaining patients were stage II and above, with stage IIB being the most common (63.9%) followed by IIIB (28.96%). The majority of the patients underwent concurrent chemoradiation (85.7%) followed by brachytherapy boost(72.4%), which is the standard of care in locally advanced cervical cancer. Most of the patients (98.1%) completed treatment without any interruption. The five-year overall survival for all stages combined was 64.52%; the majority of patients were stage II and III (92.86%), this survival is predominantly reflective of survival in these stages.

As per 2018 FIGO statistics, the stage-wise five-year survival in carcinoma cervix is as follows: stage I - 85.6% (84.1-87.0), stage II - 56.1% (51.4-60.5), stage III - 39.3 (33.9-44.7), stage IVA - 24% (13.7-15.8) [22]. The five-year survival for stage II and III combined reported in our study was slightly better compared to the FIGO report; however, it is difficult to make an exact stage-wise comparison as the number of patients in different stages were heterogenous with stage I and stage IVA having only 1.6% and 0.6% patients, respectively. Patients with stage III and above had worse overall survival, DFS, and locoregional failure-free survival than lower stages on univariate analysis but were non-significant on multivariate analysis. Besides the stage, other factors like nodal involvement [23-25], lymphovascular space involvement [26], human papillomavirus (HPV) status [27], the bulk of disease, and other pre-treatment and treatment-related factors have also been shown to affect prognosis. While the impact of some of these factors has been substantiated and, thus, incorporated into FIGO staging, the impact of others remains a subject of further research. 

Haensgen et al. [28] reported that a pre-treatment haemoglobin level >/= 11 gm/dl had better three-year survival and was an independent predictor of local failure-free survival. In another study by Thakur et al. [29], the effect of mean weekly haemoglobin (MWH) on complete response (CR) rate was analysed in patients receiving accelerated radiotherapy versus concurrent CRT. The complete response rate was significantly higher in patients receiving accelerated radiotherapy with MWH > 11 gm/dl versus those with MWH = 10-10.9 gm/dl. Meanwhile, MWH did not affect CR rates in patients on CRT, implying that chemotherapy may overcome the ill effects of anaemia on the response to treatment. In our study, a cut-off of 10 gm/dl was taken, as due to poor general condition, most patients present with haemoglobin levels < 11 gm/dl; further, it is difficult to maintain haemoglobin levels > 11 gm/dl during treatment. Thus, as an institutional protocol, a haemoglobin level >/= 10 gm/dl is aimed for by iron supplementation and blood transfusions during and before initiating treatment. In this study, pre-treatment haemoglobin level was not found to impact any of the endpoints significantly; however, the majority of patients (85.7%) received concurrent chemotherapy, which might have mitigated the effect of anaemia, as outlined above [28].

The involvement of lymph nodes was associated with worse overall survival in a study [30]. Liu et al., in a retrospective analysis of 192 stage IIB-IVA patients, found paraaortic nodal involvement to be associated with worse overall survival and progression-free survival [30]. On univariate analysis, we found pelvic and paraaortic nodal involvement associated with worse locoregional failure-free survival and DFS; however, on multivariate analysis, only paraaortic nodal involvement was significantly associated with worse disease-free survival (p-value = 0.045). As patients were treated with conventional radiotherapy, no boost dose could be given to lymph nodes or parametrium.

The impact of unilateral versus bilateral parametrial involvement was also analysed as bilateral parametrial involvement implies greater tumour bulk and, thus, poorer response to concurrent CRT, higher lymph node involvement, and more difficulty in EBRT and brachytherapy dose coverage. However, it may not always be valid, as in minimal bilateral parametrial involvement. Bilateral parametrical involvement was independently associated with worse locoregional failure-free survival (p-value = 0.020), overall survival (p-value = 0.039), and DFS (p-value = 0.039). In a FIGO reappraisal study by Lanciano et al. [31], stage IIB patients with bilateral parametrical involvement had worse four-year survival (52%) versus unilateral (70%) involvement (p-value = 0.001). For in-field local control, patients with unilateral disease had better local control (83%) versus bilateral (60%) parametrial disease (p-value = 0.001). An analysis of the medial versus lateral half of the involvement of parametrium did not show any difference in overall survival; however, it was significantly associated with better in-field local control (p-value = 0.01). Similarly, in stage IIIB patients, bilateral parametrial involvement was associated with worse survival (p-value = 0.007) and poor in-field local control (p-value = 0.04), although the difference was non-significant on multivariate analysis. Perez et al. [32] also reported worse pelvic failure rates with bilateral parametrial involvement compared to unilateral involvement in stage III disease (p-value = 0.002). In our study, stagewise subset analysis could not be done for bilateral parametrial involvement due to smaller sample sizes in subsets.

Total treatment time significantly predicts survival and pelvic control with treatment duration < eight weeks, conferring superior outcomes [33-35]. In addition, TTI has also been shown to affect survival in carcinoma cervix patients. Chen et al. [36] showed a delay of TTI resulted in a 1.33 times higher risk of death than in those who received treatment within 90 days, and patients treated after 90 days of diagnosis had lower survival compared to those treated within 90 days. In a study by George et al. [37], the early and late-stage cervical cancer patients treated within 90 days of diagnosis had a lower risk of mortality compared with those treated after 90 days. Factors shown to be associated with prolonged TTI are older age (> 75 years) [38], long distance to a treatment facility (> 100 km) [35], prolonged time to the first visit to the cancer centre, advanced stage, chemoradiation treatment, adequate treatment [39], severe comorbidity, and diagnosing hospital level at the non-medical centre or public hospital ownership [36]. Presently, there is no well-defined cut-off for TTI. Shimles et al. [40] undertook an evaluation of the association between delayed TTI and survival in cervical cancer patients, and results will be reported as a function of the four-week delay in TTI and the corresponding hazard ratio. In our study, the median time to treatment initiation was 14.7 days (range: 2.8-310.8 days), which is much less than reported in previous studies [36-39]. Eighty per cent of patients were started on treatment within 28 days of presentation to our outpatient department. There was no statistically significant difference in outcomes between those initiated on treatment within 28 days of the first visit to the OPD, versus those started after 28 days. The time interval from the initial onset of symptoms to the first visit to our institution was not available for evaluation, and thus, the delay in treatment initiation from symptom onset could not be assessed. 

Neumeyer et al. [41], in a retrospective study of 14,528 patients, reported younger women to have higher five-year survival compared with older women, 76.7% versus 46.9%, respectively. Contrary to these findings, in this study, younger age was associated with worse locoregional failure-free survival (p-value = 0.005) and DFS (p-value = 0.031). This finding was unusual but could be explained as 33.5% of patients < 50 years presented with stage IIIA or above, and only 3.5% of patients > 50 years presented with stage >/= IIIA. Thus, the advanced presentation stage could be responsible for the significantly worse effect of young age on prognosis. The late presentation of young patients could be due to a neglect of bleeding symptoms, which may be brushed aside because of irregular menstrual cycles, which may in turn be reflective of a lack of awareness among rural Indian women. Another reason could be tumour biology. In breast cancer [42,43] and colon cancer [44], younger age of diagnosis has been associated with more aggressive tumour biology and late-stage presentation. However, no such data has been reported for carcinoma cervix so far.

In several studies, non-squamous histology has been associated with poor overall survival, DFS, and locoregional failure-free survival [29,30]. Brambs et al. [45] studied the effect of tumour grade in 467 stage IB-II cervical cancer patients. The patients with poorly differentiated tumours had worse recurrence-free survival (p-value = 0.008) and overall survival (p-value = 0.031) compared with low and intermediate-grade tumours clubbed together. No relationship with lymph node involvement could be identified. However, in our study, histology and tumour grade did not significantly impact overall survival, locoregional failure-free survival, and DFS. Older patients presented more frequently with non-squamous-cell carcinoma (9.1% versus 3.7%). The frequency of poorly differentiated tumours was similar between older and young patients, 11.7% versus 10.6%.

Cervical carcinomas associated with HPV infection were shown to be associated with better overall survival and disease-free survival in a meta-analysis of 2838 patients by Lei et al. [27]. Another study by Li et al. [46] of 2845 patients studied the effect of HPV infection survival and found 43% lower excess mortality in the HPV-positive group compared to the HPV-negative group. In our study, the HPV status of the patients was not available for prognostic evaluation due to a lack of resources.

Delivering an adequate dose to the tumour bed and at-risk areas is crucial to tumour control. Brachytherapy deposits high radiation dose in the centre of the gross disease with rapid dose fall-off, sparing surrounding organs at risk [47,48]. Another means of boosting the gross tumour is supplementing EBRT techniques but used sparingly in cases where brachytherapy is not feasible. In our patients, the conventional EBRT boost technique was used in 26.4% due to the non-availability or non-affordability of brachytherapy, as elaborated previously. Patients who received an EBRT boost or had a lower total BED delivered due to organ-at-risk dose constraints and had worse DFS (p-value < 0.001), overall survival (p-value < 0.001), and locoregional failure-free survival (p-value < 0.001) on univariate analysis. However, this was non-significant in multivariate analysis. In resource-constrained settings where brachytherapy facilities are unavailable, augmenting radiotherapy with external beam radiotherapy may be contemplated. However, it's worth noting that tumour control and survival rates achieved through this method fall significantly short of those attained with brachytherapy.

In this study, several factors besides the stage of disease were found to be significant contributors to the prognosis of the disease. Identifying these factors may aid in tailoring treatment, offering more aggressive treatment to those at higher risk of recurrence. Lindegaard et al. [49] developed a tool incorporating the extent of disease per FIGO definitions using clinical drawings and MRI studies. A total T-score was calculated depending on the number of anatomical sites and sequence of involvement. This T-score was found to be highly significant for both survival and local control and outperformed FIGO staging. In the era of individualised treatment, a prognostic tool is much needed in cervical cancer for risk-wise stratification and management.

The limitation of this study was its retrospective nature. The impact of prognostic factors on different subsets could not be assessed due to the limited number of patients in the subsets. The benefit of chemotherapy could not be assessed due to fewer patients in the radiotherapy-alone arm. All patients were treated with conventional radiotherapy and 2D brachytherapy, whereas 3D CRT and image-guided brachytherapy are the present standard of care. The effect of HPV on prognosis could not be evaluated due to non-availability of testing. However, despite these limitations, this study had several strengths, such as a large sample size and availability of long-term follow-up data. Also, this study showcases patterns of care and outcomes among cervical cancer patients from rural India, who receive treatment in basic resource setups with limited facilities for diagnoses as well as treatments, which is reflective of outcomes in third-world countries such as India. The findings of this study also highlight the need to raise awareness among young women and enhance screening efforts for rural women.

## Conclusions

Young age, paraaortic nodal involvement, and bilateral parametrial involvement were independent predictors of worse prognosis for carcinoma cervix patients. Screening and counselling of young women may help in early detection and better outcomes in this age group. The development of prognostic tools or scoring systems for carcinoma cervix remains a subject of future research.
